# Controlling nutritional status score predicts 2-year outcomes in elderly patients admitted for acute heart failure

**DOI:** 10.1007/s11739-023-03230-x

**Published:** 2023-03-20

**Authors:** Davide Agnoletti, Guido Arcaro, Giuliana Scaturro, Emanuela Turcato, Elisa Grison, Elena Ferrari, Stefano Bonapace, Giovanni Targher, Filippo Valbusa

**Affiliations:** 1 Internal Medicine Department, IRCCS Sacro Cuore-Don Calabria Hospital, Negrar, Italy; 2 IRCCS Azienda Ospedaliero-Universitaria Di Bologna, Bologna, Italy; 3 Cardiovascular Internal Medicine, Department of Clinical and Surgical Sciences, University of Bologna, Via Albertoni 15, 40138 Bologna, Italy; 4 Geriatrics Department, IRCCS Sacro Cuore-Don Calabria Hospital, Negrar, Italy; 5 Division of Cardiology, IRCCS Sacro Cuore-Don Calabria Hospital, Negrar, Italy; 6 Section of Endocrinology, Diabetes and Metabolism, Department of Medicine, University and Azienda Ospedaliera Universitaria Integrata of Verona, Verona, Italy

**Keywords:** Malnutrition, CONUT score, Heart failure, Prognosis, Elderly

## Abstract

**Background:**

Heart failure (HF) is a major cause of death among the elderly. Its prevalence increases dramatically with age. The prevalence of malnourished subjects is high in hospitalized elderly patients. We aimed to investigate the prognostic role of malnutrition, assessed by controlling nutritional status (CONUT) score, on adverse clinical outcomes in the elderly admitted for acute HF.

**Methods:**

We enrolled 293 patients (mean age 84 years; 48% men) consecutively admitted for acute HF to the Internal Medicine or Geriatrics Divisions at the ‘IRCCS Sacro Cuore-Don Calabria’ Hospital of Negrar (Verona, Italy) from 2013 to 2015. We predicted the risk of all-cause death, re-hospitalizations for HF and non-HF causes, and the composite of all-cause death or hospitalizations over 2-year follow-up. Patients were divided into four groups according to CONUT score: normal-CONUT (0–1; *n* = 30); mild-CONUT (2–3; *n* = 56); moderate-CONUT (4–7; *n* = 171); and severe-CONUT (≥ 8; *n* = 36).

**Results:**

Higher CONUT scores were associated with older age and lower entry blood pressures. No difference in hemodynamics was noted at the discharge. Kaplan–Meier curves showed a significant association between worsening CONUT scores and risk of all-cause death (*p* < 0.01), re-hospitalizations (*p* < 0.01), or both (*p* < 0.001). Cox regression analysis revealed these significant associations persisted after adjustment for age, sex, pre-existing cardiovascular disease, diabetes, chronic kidney disease, heart rate, systolic blood pressure, and plasma brain natriuretic peptide levels at discharge (all-cause mortality HR = 1.29 (1.00–1.66), *p* = 0.049; hospitalization for HF HR = 1.36 (1.03–1.81), *p* = 0.033; hospitalization for non-HF HR = 1.38 (1.03–1.86), *p* = 0.034; composite outcome HR = 1.33 (1.07–1.64), *p* = 0.01).

**Conclusions:**

Malnutrition, assessed by the CONUT score, is common among elderly patients admitted for acute HF and is strongly related to increased long-term risk of all-cause death and re-hospitalizations.

## Introduction

Acute heart failure (HF) is a major and rapidly growing condition responsible for several million hospitalizations worldwide [1]. HF is also a major cause of death among the elderly in many countries [1]. The prevalence of HF increases dramatically with age, doubling approximately every 10 years in men and every 7 years in women past the age of 55 years [2]. Despite recent advances in diagnosis and management, average outcomes remain poor. Recent data from the ESC-HF pilot study showed that 1-year all-cause mortality and hospitalization rates for hospitalized HF patients were very high (17% and 44%, respectively) [3]. Malnutrition is a very common condition among patients with HF and may result from a variety of mechanisms, such as low nutritional intake due to intestinal edema and anorexia [4], liver dysfunction [5], cytokine-induced hypercatabolism [6], insulin resistance, and other mechanisms [7]. Malnutrition is one of the most relevant conditions that may adversely affect the health of older people. The prevalence of subjects with malnutrition is high among hospitalized elderly patients [8]. Some studies suggested that undernourishment, assessed by various clinical scores, may also affect clinical outcomes in middle-aged and elderly patients hospitalized for acute HF [9]. Controlling nutritional status (CONUT) score, originally proposed by Mancha et al. [10], is a widely used and validated screening tool for assessing the risk of malnutrition in hospitalized patients. This score considers serum albumin, total cholesterol, and lymphocyte count, but not body weight or other anthropometric measurements, which may be affected by congestion in decompensated patients. Recently, the CONUT score has been shown to present the most powerful predictive values on the post-discharge incidence of cardiac events, as compared to other widely used scores, and was the only score independently associated with post-discharge cardiac events [11]. To the best of our knowledge, there are no longitudinal studies that have assessed the prognostic role of the CONUT score on the long-term risk of all-cause mortality and re-hospitalizations in very elderly patients admitted for acute HF.

## Methods

### Study design

This observational prospective study aims to investigate the clinical and biological characteristics and the outcomes of elderly patients (older than 65 years), consecutively admitted to the Internal Medicine or Geriatrics Divisions at the ‘IRCCS Sacro Cuore Don Calabria’ Hospital of Negrar (Italy) with a diagnosis of acute HF. For the present analysis, data of patients enrolled from 2013 to 2015 were retrieved. All patients were initially eligible for the study if they had a confirmed clinical diagnosis of acute HF (either pre-existing or de novo congestive HF). In agreement with the 2012 European Society of Cardiology (ESC) guidelines [12], the clinical diagnosis was based on the presence of typical signs and symptoms of acute HF (e.g., breathlessness, orthopnea, paroxysmal nocturnal dyspnea, acute pulmonary edema, jugular venous distension, peripheral edema), increased brain natriuretic peptide (BNP) levels, as well as radiographic findings (e.g., pulmonary congestion/edema and cardiomegaly). We excluded from the analysis the patients who died during hospitalization, as the goal of the study was to investigate the long-term (post-discharge) outcomes. Patients lost to follow-up were considered as censored.

### Outcomes definition

The primary outcome of this study was the post-discharge all-cause mortality, and secondary outcomes were re-hospitalizations for either HF or non-HF causes; finally, we also analyzed a composite outcome, inclusive of both all-cause mortality and re-hospitalizations. Except for those who died during the first hospital admission, information on vital status (alive or deceased) and re-hospitalizations after the hospital discharge was determined by follow-up up to November 1, 2017 for each patient by phone contacts or contacts with family members or contacting the patient's physician. The mean follow-up period of the study was 2.1 years (range: 0–4.8 years). Cardiovascular disease was defined as the history of at least one of the following diagnoses: coronary heart disease, peripheral artery disease, and stroke. The date of the clinical events was registered. The study complies with the Declaration of Helsinki, the ethics committee approved the research protocol, and informed consent was obtained from the subjects (or their guardians).

### Clinical and laboratory data

Body mass index (BMI) was measured as kilograms divided by the square of height in meters. While the patient was lying in the bed since at least 10 min, blood pressure was measured with a standard aneroid sphygmomanometer. Patients were considered to have hypertension if blood pressure was ≥ 140/90 mmHg and/or if they were taking any anti-hypertensive agents. Venous blood samples were drawn on admission to the emergency department and in the morning after an overnight fast before discharge. Circulating levels of serum albumin, lipids, total lymphocyte count, creatinine (measured using a Jaffé rate-blanked and compensated assay), BNP, and other biochemical blood measurements were determined by standard laboratory procedures in the central laboratory of the hospital for all patients. Glomerular filtration rate (eGFR) was estimated by the CKI-EPI equation [13]. We calculated the CONUT score based on serum albumin, total cholesterol, and total lymphocyte count as proposed by Mancha [10]. According to the CONUT score, we divided the population into four groups: where malnutrition was absent (score 0–1), mild (2–3), moderate (4–7), or severe (8–12). As there is no standardized cutoff in literature for this specific aim, we arbitrarily chose the cutoff checking the score distribution.

Presence of coronary heart disease (CHD) was defined as a documented history of myocardial infarction, angina, or coronary revascularization procedures. The presence of chronic obstructive pulmonary disease (COPD) was confirmed by reviewing medical records of the hospital, including diagnostic symptoms patterns, and results of lung function tests. The diagnosis of permanent atrial fibrillation was made on the basis of standard electrocardiograms and medical history (reviewing hospital and physician charts from all patients). Chronic kidney disease (CKD) was defined as the presence of eGFR < 60 ml/min/1.73m^2^ [14].

### Conventional echocardiography

Conventional transthoracic echocardiography was performed by experienced cardiologists (blinded to the patients' clinical details) for measuring left ventricular (LV) diameters and wall thicknesses according to international standard criteria [15]. LV end-diastolic and end-systolic volumes and ejection fraction at rest were measured at the apical four-chamber and two-chamber views (by modified Simpson rule) [15].

### Statistical analysis

Patients were divided into four groups according to the CONUT score. Data were presented as mean values and SD for normal variables and median and interquartile range (IQR) for non-normal variables. Number and percentage were used for categorical variables. Missing data were not inferred. Differences between the four groups were tested by the one-way ANOVA for normal variables and the Kruskal–Wallis test for non-normal variables. For categorical variables, the Chi-squared test was performed. Time-to-event data are presented graphically using the Kaplan–Meier curves for each clinical outcome (all-cause death, hospitalizations for HF or non-HF causes, or both). Log-rank tests were used to compare survival and hospitalization rates among the groups. Unadjusted and adjusted Cox proportional hazard regression models were used to determine the risk for clinical outcomes according to worsening CONUT scores. These models were adjusted for age, sex, pre-existing cardiovascular disease, diabetes, chronic kidney disease, heart rate, systolic blood pressure, and plasma BNP levels at the hospital discharge. These covariates were chosen on the basis of either clinical indication or significant univariate association with each clinical outcome. The post hoc power for the primary outcome analysis, given *n* = 293, HR = 1.56, event probability = 0.48, was calculated at 75%. Statistical analyses were performed with the software R 3.3.1. *P* values < 0.05 were considered statistically significant.

## Results

**Fig. 1:**
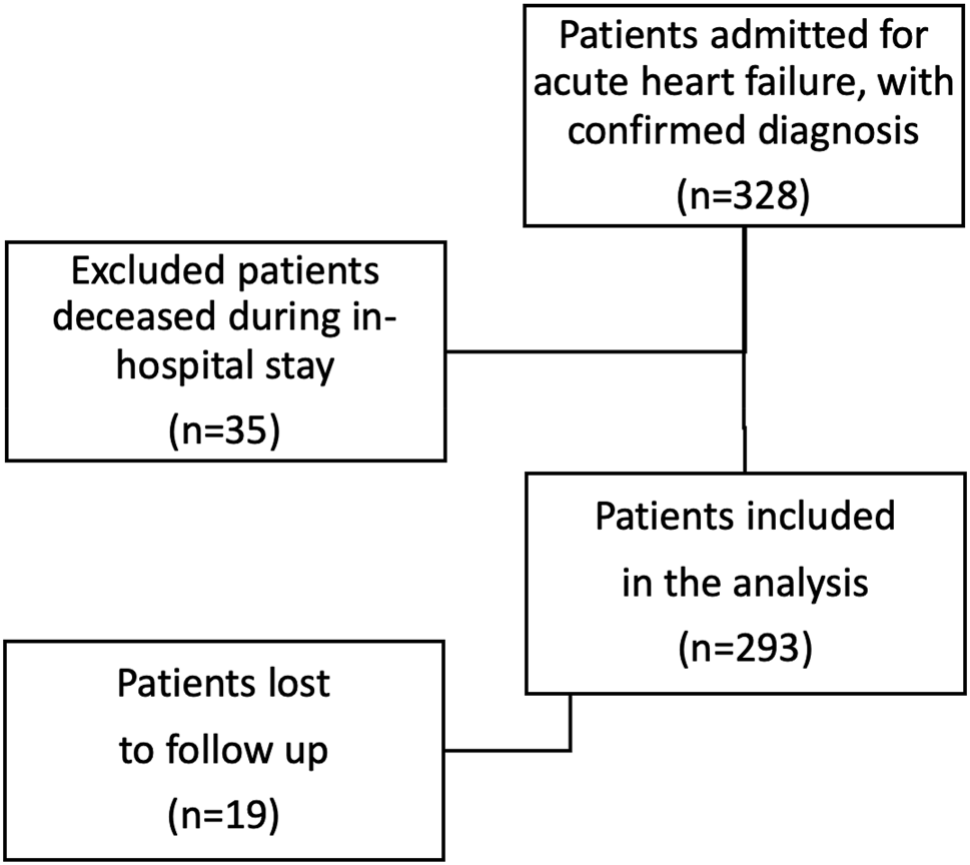
Participant flowchart

The participant flowchart is shown in Fig. 1. Of the 328 patients admitted for acute HF during the specified timeline, 35 were excluded as they died during the in-hospital stay. Nineteen patients were excluded as they were lost to follow-up. The final sample included 293 patients (141 men) and was divided into four categories according to the CONUT score: 30 subjects in the first category (score 0–1; normal nutrition); 56 in the second category (score 2–3; mild malnutrition); 171 in the third category (score 4–7; moderate malnutrition); and 36 in the fourth category (score 8–12, severe malnutrition). Mean age (± SD) of the whole sample was 83.7(7.4) years. Mean (SD) CONUT score was 4.7(2.5); 207 (70.6%) patients had moderate-to-severe malnutrition (i.e. CONUT score ≥ 4).

Table 1 shows the baseline clinical and biochemical characteristics of participants. There was a significant increase of age across the four groups, with older subjects among those belonging to the worse CONUT score. Patients with worse CONUT score also had significantly lower values of hemoglobin, triglycerides, and sodium compared with those with normal-CONUT score. As expected, they also had lower serum albumin, total lymphocyte count, and total cholesterol levels than patients in lower CONUT score categories. No significant differences were found in sex distribution, adiposity measures (body weight and BMI), and serum creatinine levels across the groups. At hospital admission, patients with lower CONUT score had lower BNP levels than patients with higher CONUT score (min 525[IQR 329–808], max 748[IQR 427–1282], pg/mL), but this trend did not achieve statistical significance (*p* = 0.09); however, at hospital discharge, plasma BNP levels were essentially comparable among the groups (ranging from 338[IQR 191–472] to 491[IQR 210–1083], pg/mL).

**Table 1:** Baseline clinical and biochemical characteristics of patients grouped by the CONUT score

	CONUT 0–1 *N* = 30	CONUT 2–3 *N* = 56	CONUT 4–7 *N* = 171	CONUT 8–12 *N* = 36	*P* value
Age, years	81.1 (9.47)	81.62 (7.34)	84.8 (6.65)	83.64 (8.28)	**0.0076**
Male sex, *n* (%)	14 (46.7)	27 (48.2)	82 (48.0)	18 (50.0)	0.99
Body weight 1, kg	76 (66–88.2)	73 (63–84)	73 (61–85)	69.5 (59–82)	0.38*
Body weight 2, kg	74.5 (64.2–87)	71 (60–79)	68 (57–79)	63.5 (56.2–75.5)	0.07*
BMI, kg/m^2^	26.7 (24.4–28.9)	26.6 (23.8–29.8)	26.2 (22.6–29.3)	24.7 (20.9–27)	0.13*
Total cholesterol, mmol/L	4.99 (0.83)	4.28 (1.07)	3.45 (0.74)	2.99 (0.93)	** < 0.0001**
Triglycerides, mmol/L	1.2 (0.9–1.5)	1.1 (0.9–1.5)	1.0 (0.8–1.2)	0.9 (0.7–1.3)	0.0024*
Lymphocyte count, × 10^9^/L	2.15 (0.61)	1.69 (0.65)	1.28 (0.49)	0.91 (0.19)	** < 0.0001**
Hemoglobin, g/dL	13.39 (1.47)	12.14 (1.88)	11.74 (2.02)	11.84 (2.04)	**0.0004**
Creatinine, umol/L	87.5 (69.5–119.5)	102.5 (79.5–143.2)	107 (82.5–140)	100 (82.8–132.2)	0.098*
eGFR, mL/min per 1.73m^2^	60.5 (22.3)	51.5 (22.6)	48.1 (20.3)	53.7 (22.9)	**0.0238**
Albumin, g/L	37.75 (2.22)	35.27 (2.38)	32.02 (3.15)	27.03 (2.28)	** < 0.0001**
Sodium 1, mmol/L	136.7 (4.15)	138.18 (3.91)	136.49 (5.17)	134.5 (7.52)	**0.0040**
Sodium 2, mmol/L	138.5 (3.6)	138.64 (2.82)	138.12 (3.78)	137.03 (4.19)	0.0841
BNP 1, pg/mL	524.5 (329–807.8)	618 (323–1156)	748 (427–1282)	688 (460–1511)	0.0973*
BNP 2, pg/mL	338 (191.2–471.5)	423 (182.2–634)	440 (252–799.5)	491 (210–1083)	0.1772*
CONUT score, AU	0 (0–1)	2 (2–3)	5 (4–6)	9 (8–9)	ND

Data are expressed as means ± SD, medians and IQR or percentages. “1” and “2” after the variable name stand for the values measured at hospital admission and discharge, respectively. *BMI* stands for body mass index, *BNP* brain natriuretic peptide, *ND* not determined

Bold font in *p* values column stands for statistical significance

^*^Not normally distributed variables, for which the Kruskal–Wallis test was used

In Table 2, data show a not significant trend toward increasing prevalence of both previous HF (from 13 to 33%) and cardiovascular disease (from 20 to 47%) across the CONUT score categories. The four groups did not significantly differ in terms of pre-existing atrial fibrillation, diabetes, CKD, peripheral artery disease, and stroke. Patients with lower CONUT score were more likely to be treated with ACE inhibitors or sartans (70%) than those with higher CONUT score (42%).

**Table 2:** Comorbidities and drug treatments of patients grouped by the CONUT score

	CONUT 0–1 *N* = 30	CONUT 2–3 *N* = 56	CONUT 4–7 *N* = 171	CONUT 8–12 *N* = 36	*P* value
	*N *(%)	*N* (%)	N (%)	*N* (%)	
Previous heart failure	4 (13.3)	7 (12.5)	41 (24.0)	12 (33.3)	0.06
Coronary heart disease	25 (83.3)	50 (89.3)	155 (90.6)	27 (75.0)	0.06
Atrial fibrillation	16 (53.3)	32 (57.1)	114 (66.7)	18 (50.0)	0.16
Diabetes mellitus	8 (26.7)	20 (35.7)	63 (36.8)	12 (33.3)	0.75
Peripheral artery disease	1 (3.3)	5 (8.9)	16 (9.4)	3 (8.3)	0.75
Chronic kidney disease	14 (46.7)	32 (57.1)	101 (59.1)	20 (55.6)	0.65
Stroke	2 (6.7)	1 (1.8)	4 (2.3)	2 (5.6)	0.45
Cardiovascular disease	6 (20.0)	19 (33.9)	60 (35.1)	17 (47.2)	0.15
*Drug treatments at hospital admission*					
ASA/clopidogrel	12 (40.0)	21 (37.5)	55 (32.2)	16 (44.4)	0.48
Beta-blockers	15 (50.0)	42 (75.0)	115 (67.3)	24 (66.7)	0.14
ACE inhibitors/sartans	21 (70.0)	31 (55.4)	77 (45.0)	15 (41.7)	**0.0450**
Anti-aldosterone drugs	13 (43.3)	18 (32.1)	59 (34.5)	14 (38.9)	0.72
Furosemide	30 (100)	56 (100)	166 (97.1)	35 (97.2)	0.47
Furosemide, daily dose*	94 (136)	89 (130)	97 (131)	86 (165)	0.12
Ivabradine	2 (6.7)	1 (1.8)	5 (2.9)	2 (5.6)	0.56
Statins	6 (20.0)	19 (33.9)	41 (24.0)	12 (33.3)	0.30

*in milligrams, mean (SD)

In Table 3 (supplemental file), data on hemodynamics are shown. There was no difference among the four groups concerning LV-ejection fraction. Mean (SD) LV-ejection fraction was 46.9% (14.2%), with a range from 10 to 78%. Patients with LV-ejection fraction < 30% were 15.2%. Systolic and diastolic BP values at hospital admission were higher in patients with the lowest CONUT score (ranging from 137/80 mmHg to 118/68 mmHg, *p* < 0.001 for trend). Patients with the highest CONUT score at hospital admission tended to have lower pulse pressure and heart rate than other patients. At discharge, patients did not show any significant difference in the hemodynamic pattern.

**Table 3:** Baseline hemodynamic parameters of patients grouped by the CONUT score

	CONUT 0–1 *N* = 30	CONUT 2–3 *N* = 56	CONUT 4–7 *N* = 171	CONUT 8–12 *N* = 36	*P* value
LV-ejection fraction	45.04 (15.76)	46.44 (16.31)	47.31 (13.51)	47.04 (13.62)	0.90
Systolic blood pressure 1	136.67 (22.18)	139.73 (21.24)	132.02 (24.66)	117.75 (19.98)	**0.0001**
Diastolic blood pressure 1	80.27 (12.87)	79.12 (13.71)	74.51 (12.6)	68.11 (10.16)	**0.0002**
Pulse pressure 1	56.4 (17.92)	60.61 (18.13)	57.51 (20.12)	49.64 (15.23)	0.06
Heart rate 1	82 (62.8–93.8)	83 (70–101)	82 (70.5–97.5)	73.5 (65.8–90)	0.26*
Systolic blood pressure 2	119.5 (13.73)	119.73 (15.21)	118.71 (15.69)	115 (17.44)	0.36
Diastolic blood pressure 2	70.17 (8.76)	68.84 (8.69)	67.95 (7.79)	66.39 (8.59)	0.38
Pulse pressure 2	49.33 (12.98)	50.89 (13.59)	50.75 (15.35)	48.61 (13.71)	0.87
Heart rate 2	70.43 (13.48)	73.39 (13.58)	74.67 (13.02)	70.28 (13.42)	0.17

Data are expressed as means ± SD. “1” and “2” after the variable name stand for the values measured at hospital admission and discharge, respectively

Bold font in *P* values column stands for statistical significance

^*^Non-normal variables, for which Kruskal–Wallis test was done

**Fig. 2:**
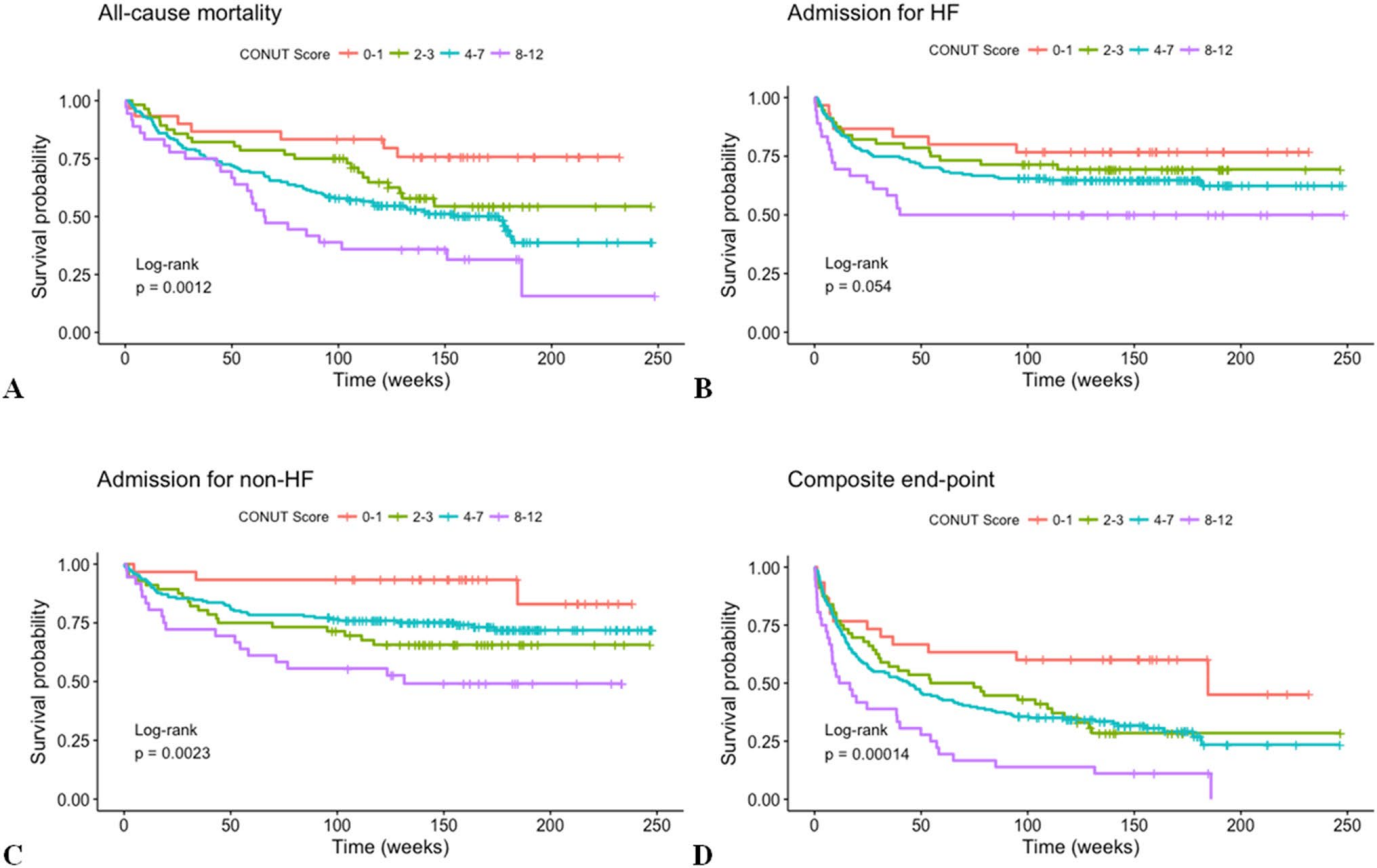
Kaplan–Meier (KM) curves for 2-year survival rates [**A**]; survival free from 2-year risk of re-hospitalizations for heart failure (HF) [**B**]; survival free from 2-year risk of re-hospitalizations for non-HF causes [**C**]; and survival free from the composite outcome [**D**], by CONUT score category

Figure 2 shows the Kaplan–Meier curves for the risk of 2-year clinical outcomes stratified by the CONUT score. For global survival, survival free from re-hospitalizations for either HF or non-HF causes, and for the composite outcome, the CONUT score performed well in discriminating the population at risk. The primary outcome occurred in 142 of 293 patients (48.5%); the hospitalization outcome occurred in 103 (35.2%) and 85 (29%) patients considering HF and non-HF causes, respectively; the composite outcome occurred in 204 patients (69.6%). The outcome rates per 100 person-year were, respectively, 23.35, 16.36, 11.64, and 51.11.

Table 4 shows significant hazard ratios for each of the clinical outcomes calculated for each increment in one CONUT category, both in the unadjusted and adjusted Cox regression models, where the covariates were: age, sex, cardiovascular disease, diabetes, chronic kidney disease, systolic blood pressure, plasma BNP levels, and heart rate at hospital discharge.

**Table 4:** Cox regression models. Association between increasing CONUT scores and 2-year risk of adverse clinical outcomes

Outcome	Unadjusted			Adjusted*		
	Hazard Ratio	95% CI	*P* value	Hazard Ratio	95% CI	*P* value
All-cause mortality	1.56	(1.24–1.95)	** < 0.001**	1.29	(1.00–1.66)	**0.049**
Hospitalization for HF	1.39	(1.07–1.80)	** < 0.001**	1.36	(1.03–1.81)	**0.033**
Hospitalization for non-HF causes	1.47	(1.10–1.96)	** < 0.001**	1.38	(1.03–1.86)	**0.034**
Composite outcome	1.44	(1.19–1.73)	** < 0.001**	1.33	(1.07–1.64)	**0.01**

*HF* stands for heart failure

^*^Adjusted for age, sex, cardiovascular disease, diabetes, chronic kidney disease, systolic blood pressure at discharge, plasma BNP levels at discharge, heart rate at discharge

The hazard ratios are calculated for each increment of the CONUT category (i.e. “0–1”; “2–3”; “4–7″;” ≥ 8″)

Composite outcome was defined as all-cause death + re-hospitalizations for either HF or non-HF causes

Bold font in *P* values column stands for statistical significance

## Discussion

In a population of very old patients (mean age > 80 years) hospitalized for acute HF, we detected four nutrition levels (defined by the CONUT score) able to discriminate the 2-year risk of all-cause death and re-hospitalizations, independently of the hemodynamic status reached during the first hospital admission. In particular, for any increment of CONUT category, indicating a worse nutrition level, we found 30% higher risk of all-cause death, 36% and 38% higher risk of re-hospitalization for HF or other causes, respectively, and 33% higher risk of the composite outcome (any of the previous ones) over a period of 2 years. The first observation is that our population was composed mainly of very old patients with a high prevalence of moderate-to-severe malnutrition (~ 70% based on the CONUT score). Indeed, malnutrition is known to be a common condition among patients with HF, mainly due to insufficient caloric intake and hypercatabolism. Patients with HF developing under-nutrition may enter the so-called MIC (malnutrition, inflammation, cachexia) vicious circle [6, 9]. Malnutrition is also a highly prevalent condition in elderly people, and many studies have shown a link between malnutrition and increased mortality risk in such population [7, 16–26]. The combination of HF and malnutrition is, therefore, a bad ‘brother’ for elderly patients. The age effect in our analysis appeared to be marginal: even if patients with worse CONUT score were older, the adjusted analyses did not show any role of age in the event risk prediction. At first hospital admission, patients in the four CONUT groups were comparable for sex distribution, the prevalence of previous cardiovascular events, and many pharmacological treatments. At discharge, patients had similar hemodynamic and laboratory characteristics, with lower body weight, higher serum sodium, lower plasma BNP, and lower blood pressure compared with values seen at hospital admission. This could be likely due to treatment success and homogeneous care of the acute HF by physicians. The survival analysis showed that 2-year rates of all-cause death and re-hospitalizations were well discriminated by the CONUT score. Indeed, our analysis revealed a significant and graded relationship between the worsening of the nutritional state and the worsening of the prognosis, no matter of the outcome, and independently of main potential confounders. Of note, Fig. 2B shows a log rank at the significance limits. This is likely due to an outlier, Mr. F. (85 years old), who did not present any acute HF after the discharge, during the follow-up period, while presenting a CONUT score of 12. Excluding the outlier from the analysis, the *p* value becomes significant (*p* = 0.037, data not shown).

In literature, the relationship between the nutritional state and prognosis has been investigated in some studies of patients with HF. In patients admitted to the intensive care unit for severe HF, Shirakabe et al. showed that malnutrition indexes were independent predictors of all-cause mortality after a 1-year follow-up [24]. Of note, these patients were in more severe clinical conditions and younger (median age 76yrs) than patients in our study. Our results are in line with data from the literature, particularly the studies by Sze et al. [25] in more than 3000 patients aged 75 years (median, 67–81 IQR), by Iwakami [18], and by Yoshihisa [26] in even younger patients (mean age 66.5 years). The study by Bermejo et al. investigated the prognostic role of the CONUT score on mortality and hospitalization [16]. In 145 patients (mean age 70yrs) admitted for HF, they found that the CONUT score predicted HF and non-HF hospitalization, but not overall mortality. A recent paper investigated the factors linked to mortality in 164 elderly patients (mean age 82.9ys) hospitalized for acute heart failure. They considered only standard biological assessment and found that lower BMI and hypoalbuminemia were correlated to a higher mortality risk [27].

Our study examined almost 300 elderly patients admitted for acute HF and found that the CONUT score, a comprehensive index of nutritional status, was able to discriminate the 2-year risk of all-cause mortality, as well as re-hospitalizations, or both. These results support the hypothesis that the CONUT score is a simple and robust method to track down the patients at higher risk of events even after 80 years, independently of common clinical risk factors. Interestingly, both HF and non-HF hospital readmissions were also strongly predicted by the CONUT score. This is in line with the notion that frail elderly patients easily enter the MIC vicious circle and are not only concerned by cardiovascular disease itself, but also by metabolic, inflammatory, and disability complications.

Our results confirm and enhance the data from Hamada et al. on 67 elderly admitted for HF [17]. Indeed, these authors found that patients with CONUT score ≥ 5 were more likely to have cardiac events, namely death or HF re-hospitalization during the follow-up. Interestingly, our data further highlight a progressive increase in the risk of cardiac and non-cardiac events among all the nutritional classes assessed by the CONUT score.

## Limitations

Our study has some important limitations that should be mentioned. Firstly, even if the clinical outcomes were prospectively collected, the analysis was made retrospectively using the patients’ files. Secondly, we could not obtain other indirect estimations of the nutritional state due to insufficient patients’ information. Thirdly, the CONUT score is only an estimate of the nutritional status, and is likewise influenced by the actual volume charge of the patients; the interpretation of the results should be therefore cautious. Finally, our cohort data were from almost 10 years ago, and HF definition and treatment relied on old guidelines. Fortunately, newer guidelines have definitively changed the treatment of HF patients with the introduction of new drug classes with important effect on mortality and HF re-hospitalization. Therefore, the conclusion of our study may not apply anymore to contemporary HF populations. However, considering that both patients with high and low CONUT score will now undergo this modern approach, it is easy to suppose that the outcome prediction will not change. Anyway, to solve this issue, we plan in the near future to implement a new analysis on the most recent database to get new insight into the predictive value of the CONUT score.

The main strengths of this study are that (i) we deal with a very old population of patients with HF, with a relatively large sample size; (ii) we collected information about cardiac and non-cardiac outcomes, with a follow-up of 2 years; (iii) we showed a significant added prognostic value of the CONUT score in the long-term risk prediction of all-cause death and re-hospitalizations in patients with HF.

## Conclusions

The results of this longitudinal study show that malnutrition, as assessed by the CONUT score, is common among elderly patients admitted for acute HF and is able to strongly predict the cardiac and non-cardiac 2-year prognosis 2 years after the discharge. Indeed, a prompt and comprehensive medical and nutritional care of these patients, drawn to prevent or even reverse poor nutritional status, could lead to an improvement of cardiac and non-cardiac prognosis [19–23].

## Data Availability

The datasets generated during and/or analyzed during the current study are available from the corresponding author on reasonable request.
